# MUC5B rs35705950 and its association with survival in Brazilian patients with idiopathic pulmonary fibrosis: A longitudinal cohort study

**DOI:** 10.1371/journal.pone.0341661

**Published:** 2026-01-29

**Authors:** Rogerio Rufino, Luana Faria, Marcelo Ribeiro-Alves, Lucas Resende Martinez Araujo, Leonardo Palermo, Elizabeth Bessa, Bruno Rangel, Mariana Carneiro Lopes, Mariana Costa Rufino, Cláudia Henrique da Costa, Jeane de Souza Nogueira, Cíntia Barros Santos-Rebouças, Luís Cristóvão Porto

**Affiliations:** 1 Rio de Janeiro State University, Rio de Janeiro, Brazil; 2 Fundacao Oswaldo Cruz, Rio de Janeiro, Brazil; 3 Idomed, Rio de Janeiro, Brazil; 4 São Paulo University, Bauru, Brazil; 5 TIXUS - Technologic Core for Tissue Repair and Histocompatibility, Rio de Janeiro State University, Rio de Janeiro, Brazil; 6 Human Genetics Laboratory (SERVGEN), Department of Genetics, Institute of Biology Roberto Alcantara Gomes, Rio de Janeiro State University, Rio de Janeiro, Brazil; Kurume University School of Medicine: Kurume Daigaku Igakubu Daigakuin Igaku Kenkyuka, JAPAN

## Abstract

**Background:**

Idiopathic pulmonary fibrosis (IPF) is a progressive interstitial lung disease with heterogeneous clinical outcomes. The MUC5B promoter polymorphism (rs35705950) is the most consistent genetic factor associated with IPF susceptibility, but data from Latin American populations remain limited.

**Methods:**

We conducted a longitudinal cohort study including 50 patients with IPF and 45 healthy controls, recruited between August 1, 2018, and April 30, 2025, at a tertiary referral center in Brazil. Genotyping of rs35705950 was performed by real-time PCR. Demographic, clinical, and functional data were collected at baseline. Survival was analyzed using Kaplan–Meier curves and Cox regression models, with follow-up defined from symptom onset to death or censoring.

**Results:**

The T-allele frequency was higher in IPF patients compared with controls (47.1% vs 8.3%, p < 0.001). Carriers of the T allele (G/T or T/T) comprised 82.4% of cases versus 16.7% of controls. Among patients with IPF, T-carriers tended to have worse lung function and shorter survival, although survival differences were not statistically significant. In multivariable models, the presence of the T allele was not an independent predictor of mortality. By contrast, higher New York Heart Association (NYHA) functional class was consistently associated with increased mortality risk.

**Conclusions:**

In this Brazilian cohort, the MUC5B promoter variant was strongly associated with susceptibility to IPF but not with independent survival differences. Functional impairment, particularly NYHA class, remained the key prognostic factor. These findings highlight the importance of integrating genetic and clinical information in diverse populations and provide novel data from an underrepresented Latin American setting.

## Introduction

Idiopathic pulmonary fibrosis (IPF) is a chronic, progressive lung disease of unknown origin that ultimately leads to respiratory failure. Histological examination typically reveals a pattern consistent with usual interstitial pneumonia (UIP). Clinically, patients often present with progressive dyspnea, functional decline, and poor prognosis [1,2]. Despite advances in antifibrotic therapies, IPF remains highly heterogeneous, with some patients experiencing rapid progression and others showing a more gradual decline [3–5]. In recent years, genetic factors have gained recognition as important contributors to both susceptibility and disease course in IPF [5,6]. One of the most extensively studied variants is the single nucleotide polymorphism (SNP) rs35705950, located in the promoter region of the *MUC5B* gene [6–9]. This polymorphism is associated with increased expression of mucin 5B in the distal airways, a phenomenon hypothesized to impair mucociliary clearance and facilitate fibrotic remodeling [10–12]. Multiple studies in European and North American populations have demonstrated a strong association between the *MUC5B* T allele and the risk of developing IPF. Its prognostic impact, however, has been less consistent, with some studies suggesting improved survival among carriers and others indicating worse outcomes [13–15]. Importantly, most evidence on rs35705950 derives from populations of European ancestry [5,16]. In contrast, data from Latin America are scarce, despite the region’s unique genetic admixture of European, African, and Indigenous ancestries [17]. This admixture may influence not only the frequency of the variant but also its effect on susceptibility and disease progression. Given this underrepresentation, the present study investigates the frequency of the *MUC5B* rs35705950 polymorphism and its association with survival in a Brazilian cohort of patients with IPF. Understanding whether this variant confers comparable susceptibility and prognostic value in an admixed population is crucial for improving risk stratification, advancing personalized medicine, and contributing to the global understanding of genetic determinants of IPF. Furthermore, integrating genetic information with clinical variables such as functional impairment and family history may provide a more comprehensive approach to prognostication.

## Methods

### Study design

This longitudinal cohort study was conducted between August 1, 2018, and April 30, 2025, at the Pulmonology Outpatient Clinic of the Piquet Carneiro University Polyclinic, Rio de Janeiro State University (PPC/UERJ), Brazil. Eligible patients were enrolled after comprehensive clinical evaluation and written informed consent. The study was approved by the Research Ethics Committee of the Pedro Ernesto University Hospital (protocol no. 2.522.585; CAAE: 82001417.9.0000.5259) and conducted in accordance with national and international ethical standards. Patients with IPF were followed for up to five years to assess clinical outcomes and overall survival.

Genetic association analyses used the full genotyped set (51 IPF/ 48 controls) to preserve information on rare genotypes. Clinical and prognostic analyses (including survival) used the final analyzed cohort (50 IPF/ 45 controls) after technical exclusions.

To minimize selection bias, we enrolled consecutive eligible patients from a single tertiary clinic and used frequency-matched controls on age and sex. Information bias was addressed by standardized clinical assessments and predefined diagnostic criteria. Genotyping quality control included call-rate checks, cluster plot inspection, duplicate concordance, and evaluation of Hardy-Weinberg equilibrium in controls. Given Brazil’s admixed population, residual confounding by genomic ancestry is possible beyond self-reported ethnicity; we therefore adjusted for ethnicity and discuss this limitation explicitly. Potential survivor/selection bias related to smoking was anticipated and exploratory associations were interpreted cautiously. To reduce immortal-time bias, survival time was anchored at symptom onset, with a sensitivity analysis using date of diagnosis as time origin.

### Study population

Inclusion criteria were age ≥ 55 years and a multidisciplinary diagnosis of IPF according to the 2022 ATS/ERS/JRS/ALAT Clinical Practice Guideline [2]. Diagnosis was based on compatible clinical features (e.g., exertional dyspnea, dry cough) and HRCT demonstrating a usual interstitial pneumonia (UIP) pattern. Other causes of pulmonary fibrosis (connective tissue disease, environmental exposures, drug toxicity) were excluded. Exclusion criteria included pregnancy and pulmonary fibrosis of known etiology (e.g., connective tissue disease, hypersensitivity pneumonitis, occupational lung disease). Controls were healthy blood-donor volunteers without chronic respiratory disease, systemic inflammatory conditions, or major comorbidities, and were frequency-matched to patients by age and sex.

### Genotyping of MUC5B rs35705950

Peripheral blood was collected in EDTA tubes, and genomic DNA was extracted using the QIAamp DNA Blood Mini Kit (Qiagen, Valencia, CA) following the manufacturer’s instructions. DNA concentration was measured spectrophotometrically at 260 nm, and samples were stored at −20 °C until analysis. Genotyping of rs35705950 was performed using TaqMan® SNP Genotyping Assays (Thermo Fisher Scientific, Foster City, CA) with VIC- and FAM-labeled probes for allelic discrimination. Each 10 µL reaction contained 40 ng DNA, 40X TaqMan SNP Genotyping Assay, and TaqMan Genotyping Master Mix. Amplification was carried out on an ABI StepOnePlus Real-Time PCR System under the following conditions: 60 °C for 30 s, 95 °C for 10 min, followed by 40 cycles of 95 °C for 15 s and 60 °C for 1 min, and a final post-PCR read at 60 °C for 30 s. Allelic discrimination was automatically determined with StepOne software.

### Sample size calculation

Sample size estimation was based on the expected difference in T-allele frequency between IPF patients and controls. Previous data indicated that 76% of patients and 18% of controls carried at least one T allele. Assuming α = 0.05, 80% power, and a 1:1 case-control ratio, the minimum sample required was 18 participants per group (36 total). To allow genotype-specific analyses (TT vs GT vs GG) and improve statistical power for subgroup comparisons, we recruited 50 patients with IPF and 50 controls. This exceeded the minimum requirement and increased the robustness of analyses, particularly for the less frequent TT genotype.

### Statistical analysis (STROBE extension)

Analyses were conducted on complete cases; the proportion of missing data per variable is summarized in Supplementary S1 Dataset and no imputation was undertaken given low missingness. For case–control susceptibility, we prespecified the dominant and allelic genetic models as primary. Because of sparse cells and complete separation (T/T = 0 among controls), we used two-sided Fisher’s exact tests for contingency analyses and fitted logistic regression models for adjusted estimates. As sensitivity analyses, we additionally fitted exact and Firth-penalized logistic models for genotype-level contrasts; these did not change the interpretation of the primary models (reported in the Supplement). For lung function (FVC and SpO₂), we used multiple linear regression adjusted for age, sex, and ethnicity; when pairwise contrasts were examined, Tukey’s HSD was applied. For time-to-event outcomes, we used Cox proportional hazards models with the time scale defined from symptom onset to death/censoring; ties were handled with the Efron method. The primary Cox model adjusted for age, sex, current smoking, and NYHA; an additive per-allele model (HR per T copy) and a diagnosis-anchored time-origin model were prespecified as sensitivity analyses. Proportional hazards were assessed by Schoenfeld residuals. All tests were two-sided with α = 0.05. Analyses used R (v4.3.1).

#### Genetic association (susceptibility).

Primary contrasts were prespecified as: genotype-level (G/T vs G/G; T/T vs G/G), dominant (G/T + T/T vs G/G), recessive (T/T vs G/G + G/T), overdominant (G/T vs G/G + T/T), and allelic (T vs G). Given sparse cells (0 T/T in controls), we prioritized dominant and allelic models and used two-sided Fisher’s exact tests; recessive contrasts were treated as not calculable under standard methods. As sensitivity, we explored exact/Firth logistic models (Supplement). Where estimable, odds ratios (OR) with 95% confidence intervals (CI) are presented. (Adjusted logistic models including age, sex, and self-reported ethnicity were prespecified; recessive contrasts were not estimable. Fully adjusted results are provided when estimable in the Supplement.)

#### Pulmonary function models.

FVC (L and % predicted) and resting SpO₂ were analyzed by multiple linear regression across *MUC5B* genotypes, adjusted for age, sex, and ethnicity. Exploratory interaction with NYHA class was assessed; marginal means were computed holding covariates at observed means/proportions. Pairwise contrasts were corrected with Tukey’s HSD where applicable.

#### Survival analysis.

The primary time origin was symptom onset; a sensitivity analysis used date of diagnosis. Follow-up extended to death or administrative censoring at April 15, 2025. Kaplan-Meier curves were compared with log-rank tests. Cox proportional hazards models estimated hazard ratios (HR) with 95% CI, adjusting for age, sex, current smoking, and NYHA (mMRC considered in secondary models). Proportional-hazards assumptions were evaluated using scaled Schoenfeld residuals and complementary diagnostics; no material violations were detected. Analyses were performed in R 4.3.1; two-sided p < 0.05 was considered statistically significant.

## Results

### Study population characteristics

A total of 99 participants were initially recruited (51 IPF; 48 controls). Four were excluded due to technical issues (1 IPF; 3 controls), yielding a final analyzed cohort of 95 participants (50 IPF; 45 controls). Median age was 69 years (IQR 10) in IPF and 68 years (IQR 9.25) in controls; women comprised 39.2% and 37.5%, respectively. IPF patients more frequently self-identified as white (84% vs 68.8%); current smoking was uncommon (2% vs 10.4%). Comorbidities were frequent (92% vs 85.4%). As expected, antifibrotic therapy (88.2%) and supplemental oxygen (35.3%) occurred exclusively among IPF patients. Baseline pulmonary function in IPF showed median FVC 2.16 L (IQR 0.91), 69% predicted (IQR 32.8), and FEV₁ 1.74 L (IQR 0.67), 76.5% predicted. Resting oxygen saturation was 96% (IQR 5.5). HRCT features consistent with fibrosis were present in 96.1% of IPF patients; 21.6% had undergone lung biopsy (Table 1).

**Table 1:** Demographic, clinical, functional and outcomes distribution between the groups.

Variable		Overall	Group		p-value
			Control	IPF	
Sex	female	38 (38.4%)	18 (37.5%)	20 (39.2%)	1
	male	61 (61.6%)	30 (62.5%)	31 (60.8%)	
Age		69 (IQR = 10)	68 (IQR = 9.25)	69 (IQR = 10)	0.891
Ethnicity	white	75 (76.5%)	33 (68.8%)	42 (84%)	0.123
	brown/black	23 (23.5%)	15 (31.2%)	8 (16.0%)	
Family History of PF		11 (21.6%)	0	11 (21.6%)	NA
Current Smoking	no	40 (40.4%)	20 (41.7%)	20 (39.2%)	0.173
	ex	53 (53.5%)	23 (47.9%)	30 (58.8%)	
	yes	6 (6.1%)	5 (10.4%)	1 (2.0%)	
Tobacco Load		10 (IQR = 30)	7.5 (IQR = 30)	10 (IQR = 35)	0.36
BMI		26.44 (IQR = 4.62)	----	26.44 (IQR = 4.62)	NA
Comorbidity		87 (88.8%)	41 (85.4%)	46 (92.0%)	0.476
OLD		12 (12.2%)	3 (6.2%)	9 (18.0%)	0.143
Velcro sign		52 (52.5%)	1 (2.1%)	51 (100%)	< 0.001
Oxygen		18 (18.2%)	----	18 (35.3%)	< 0.001
Satp		96 (IQR = 5.5)	----	96 (IQR = 5.5)	NA
NYHA	1	15 (30%)	----	15 (30.0%)	NA
	2	12 (24%)		12 (24.0%)	
	3	17 (34%)		17 (34.0%)	
	4	6 (12%)		6 (12.0%)	
mMRC	0	4 (8%)	----	4 (8.0%)	NA
	1	12 (24%)		12 (24.0%)	
	2	14 (28%)		14 (28.0%)	
	3	13 (26%)		13 (26.0%)	
	4	7 (14%)		7 (14.0%)	
FVC L		2.16 (IQR = 0.91)	----	2.16 (IQR = 0.91)	NA
FVC (%)		69 (IQR = 32.75)	----	69 (IQR = 32.75)	NA
FEV_1_ (L)		1.74 (IQR = 0.67)	----	1.74 (IQR = 0.67)	NA
FEV_1_ (%)		76.5 (IQR = 32.75)	----	76.5 (IQR = 32.75)	NA
CT		50 (50.5%)	1 (2.1%)	49 (96.1%)	< 0.001
Biopsy		11 (11.1%)	----	11 (21.6%)	0.002
Anti-Fibrotic		45 (45.5%)	----	45 (88.2%)	< 0.001
Death		46 (46.9%)	8 (16.7%)	38 (76.0%)	< 0.001

Data are n (%) or median (IQR). SpO₂ = resting peripheral oxygen saturation. Missing values per variable are listed in Supplementary Table. Abbreviation: OLD – Other Lung Disease: Asthma or COPD; PF -Pulmonary Fibrosis; FVC - Forced Vital Capacity (FVC); L = liter; FEV_1_ – Forced Expiratory Volume in 1 second; Satp – Peripheral Oxygen Saturation; NYHA – New York Heart Association Functional Classification; MRC – Medical Research Council Dyspnea Scale; IQR – Interquartile Range; NA – Not Applicable.

Genotype distribution in controls conformed to Hardy–Weinberg equilibrium (χ² = 0.397, p = 0.82). Among controls, 83.3% were G/G, 16.7% G/T, and 0% T/T; among IPF patients, 17.6% were G/G, 70.6% G/T, and 11.8% T/T (p < 0.001). The T-allele frequency was 47.1% in IPF vs 8.3% in controls.

Using the full genotyped set (51 IPF/ 48 controls), susceptibility contrasts showed: dominant model (G/T + T/T vs G/G) OR 23.30 (95% CI 8.19–66.42), p < 0.0001; allelic model (T vs G) OR 9.77 (4.29–22.23), p < 0.0001. Due to complete separation (0 T/T in controls), recessive and T/T vs G/G ORs were NC; corresponding Fisher’s p-values were 0.0270 and 0.0002, respectively (Table 2, S1 Table).

**Table 2:** Distribution of *MUC5B* rs35705950 genotypes and association with idiopathic pulmonary fibrosis (IPF).

Section	Model/Contrast	Controls (n,%)	IPF Cases (n,%)	OR (95% CI)	p (Fisher)
Genotype distribution	G/G	40 (83.33%)	9 (17.65%)	ref	
Genotype distribution	G/T vs G/G	8 vs 40	36 vs 9	20.00 (6.97–57.35)	<0.0001
Genotype distribution	T/T vs G/G	0 vs 40	6 vs 9	NC	0.0002
Genetic models	Dominant (G/T + T/T vs G/G)	8 vs 40	42 vs 9	23.30 (8.19–66.42)	<0.0001
Genetic models	Recessive (T/T vs G/G + G/T)	0 vs 48	6 vs 45	NC	0.0270
Genetic models	Overdominant (G/T vs G/G + T/T)	8 vs 40	36 vs 15	12.00 (4.55–31.62)	<0.0001
Allelic model	T vs G (alleles)	T = 8; G = 88	T = 48; G = 54	9.77 (4.29–22.23)	<0.0001

Genotype-level contrasts are shown against the reference category (G/G). Genetic models were specified as: Dominant (G/T + T/T vs G/G), Recessive (T/T vs G/G + G/T), and Overdominant (G/T vs G/G + T/T). The allelic model compares T vs G using allele counts. Odds ratios (OR) with 95% confidence intervals (CI) quantify the association with IPF; two-sided Fisher’s exact p-values are provided owing to sparse cells. Results correspond to the genotyping set reported in Supplementary. Abbreviations/Notes: ref, reference category (G/G); NC, not calculable under standard methods due to complete separation (e.g., zero T/T in controls); OR, odds ratio; CI, confidence interval; p (Fisher), two-sided Fisher’s exact test p-value; IPF, idiopathic pulmonary fibrosis. Model definitions: Dominant = (G/T + T/T) vs G/G; Recessive = T/T vs (G/G + G/T); Overdominant = G/T vs (G/G + T/T); Allelic = T vs G (allele counts). Susceptibility analyses used the 51/48 genotyped set; clinical/prognostic analyses used the 50/45 cohort.

**Fig 1:**
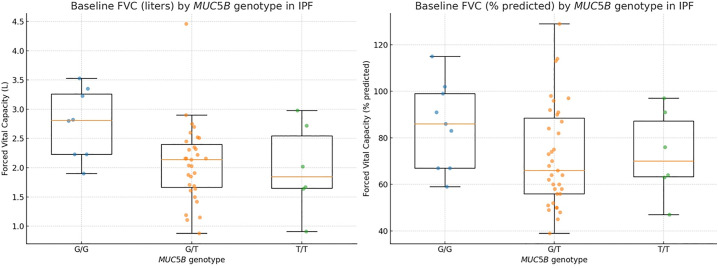
Baseline pulmonary function (FVC) according to *MUC5B* rs35705950 genotype in patients with idiopathic pulmonary fibrosis (IPF). Legend: Box-and-strip plots display forced vital capacity (FVC) in liters (A) and percent predicted (B) stratified by genotype (G/G, G/T, T/T). Central circles show marginal means from linear models adjusted for age, sex, and ethnicity, with covariates held at observed means/proportions; error bars indicate 95% CI. Pairwise comparisons were Tukey-adjusted. T-allele carriers exhibited lower FVC than G/G.

Within the IPF cohort, T-allele carriers (G/T or T/T) exhibited worse lung function at baseline than G/G homozygotes (median FVC 2.14 L vs 2.81 L; 66% vs 86% predicted), with similar trends for FEV₁ and resting SpO₂. These differences remained after adjustment for age, sex, and ethnicity (Fig 1).

**Fig 2:**
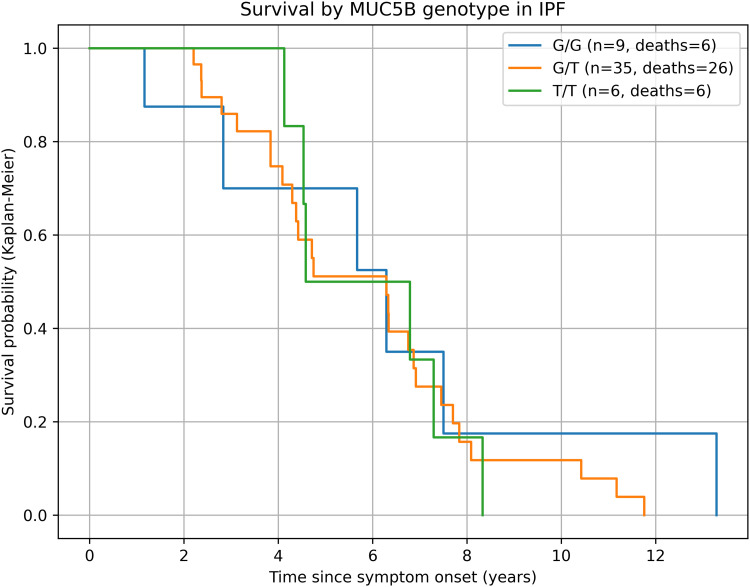
Kaplan-Meier survival by MUC5B rs35705950 genotype in idiopathic pulmonary fibrosis (IPF). Legend: Step plots show survival from symptom onset stratified by genotype. Curves suggest lower survival among T-allele carriers, although differences were not significant (overall log-rank χ² = 1.07; p = 0.30). Time in years; Numbers at risk are provided in the Supplement.

Over a maximum of five years from symptom onset, 38/50 IPF patients (76%) died. Median survival was 7.5 years (G/G), 6.9 years (G/T), and 6.8 years (T/T); grouped, T-allele carriers had 6.8 vs 7.5 years for G/G. Kaplan–Meier curves suggested lower survival among T-allele carriers, but differences were not statistically significant (log-rank χ² = 1.07, p = 0.30); pairwise log-rank tests were also non-significant. In Cox models, T-allele carriage was associated with a non-significant increase in mortality risk versus G/G (univariable HR 1.85, 95% CI 0.66–5.17; p = 0.24). In sensitivity analyses using diagnosis as time origin and an additive per-allele model, effect directions were unchanged and remained non-significant. The primary adjusted model (age, sex, current smoking, NYHA) showed a concordant but non-significant association (aHR 1.85, 95% CI 0.66–5.17). Results were similar in a sensitivity analysis using time from diagnosis (aHR 2.09, 95% CI 0.81–5.42). By contrast, NYHA class was consistently and independently associated with mortality (HR 1.50 per class, 95% CI 1.07–2.10; p = 0.018). No independent associations were observed for sex, smoking status, antifibrotic therapy, or oxygen use (Fig 2; Table 3).

**Table 3:** Multivariable Cox proportional hazards for all-cause mortality in idiopathic pulmonary fibrosis (IPF).

Variable	aHR	95% CI	p_value
T carrier	1.853	0.664-5.174	0.391
Age	0.971	0.932-1.010	0.1448
Sex	1.257	0.618-2.554	0.5278
Current Smoking	1.891	0.329-10.882	0.4756
NYHA	1.502	1.071-2.105	0.0183

Time scale: years from symptom onset to death or censoring (April 15, 2025). Models adjusted for age, sex, current smoking status, and NYHA functional class. “T-carrier” denotes presence of the rs35705950 T allele (G/T or T/T) versus G/G. Values are adjusted hazard ratios (aHR) with 95% confidence intervals; p values are two-sided. The “Comment” column summarizes the direction and statistical significance of each association. Abbreviations: aHR, adjusted hazard ratio; CI, confidence interval; NYHA, New York Heart Association functional class.

## Discussion

To the best of our knowledge, this study is among the first to examine, in a Brazilian cohort, the relationship between the MUC5B promoter variant, functional status, family history, and survival outcomes in IPF. In a setting where Latin American data are scarce, our longitudinal design and systematic follow-up provide needed regional evidence.

We found a markedly higher frequency of the minor MUC5B T allele among patients with IPF versus controls, with large effect sizes in both dominant and allelic models (OR 23.30 and 9.77, respectively). These results are directionally consistent with reports from predominantly European/North American cohorts showing a strong susceptibility signal for MUC5B rs35705950 [10,18]. The T-allele frequency in our IPF cohort (82.4%) mirrors those studies, while its frequency in Brazilian controls (16.7%) likely reflects the country’s admixed genetic background [19,20,21,22]. Self-reported ethnicity may incompletely capture genomic ancestry in an admixed population; future multicenter studies with principal components are warranted.

Mechanistically, rs35705950 sits ~3 kb upstream of the *MUC5*B transcription start site and has been linked to increased gene expression through enhancer activity and altered chromatin accessibility; higher expression is typically observed in T-allele carriers [23,24]. Increased distal airway *MUC5B* expression provides a biologically plausible pathway connecting the variant to impaired mucociliary clearance and fibrotic remodeling [25,26].

Prognostically, the literature is mixed: some cohorts reported better survival in carriers [27], whereas others suggest context-dependent or age-restricted benefits [28]. In our clinical/prognostic set, survival curves trended worse in T-carriers but were not significant (log-rank p = 0.30), and MUC5B was not an independent predictor in adjusted Cox models (aHR 1.85; sensitivity aHR 2.09). This aligns with evidence that the variant’s prognostic effect is heterogeneous across ancestry, design choices (e.g., time origin), and treatment era [29]. Treatment variables were modeled at baseline due to limited temporal granularity; time-varying effects will be important to assess in larger cohorts.

By contrast, functional impairment remained a consistent predictor: each increase in NYHA class conferred ~50% higher mortality risk in our cohort, in keeping with established clinical literature that symptom burden and physiologic limitation capture near-term risk more robustly than genotype alone [3,30]. Notably, adding genotype to clinical scores may offer modest incremental discrimination in some settings [28], but our data emphasize prioritizing functional staging in routine care.

Exploratory findings, like an inverse association between smoking and mortality, should be interpreted cautiously as potential survivor/selection bias rather than protection [3]. Beyond IPF, pleiotropy underscores context: in a large biobank analysis, the *MUC5B* T allele associated with lower post-COVID pneumonia without increasing severe COVID outcomes [31], suggesting environment-dependent effects.

Strengths of our study include strict genotyping procedures and verification of proportional hazards. This single-center design may limit generalizability across Brazil’s heterogeneous and admixed population. Although we adjusted for self-reported ethnicity, genomic ancestry may not be fully captured; multicenter studies incorporating ancestry principal components are needed to refine effect estimates. Baseline treatment variables (antifibrotics and oxygen use) were modeled as fixed covariates because detailed initiation/cessation dates were unavailable; future cohorts with time-varying treatment data will be important to disentangle therapy effects on survival. Small genotype strata (e.g., G/G and T/T) led to wide confidence intervals and separation in some contrasts; we therefore prioritized dominant and allelic models and confirmed robustness via exact/Firth sensitivity analyses. Finally, incomplete availability of D_L_CO/6MWD limited comparison with GAP-based prognostic frameworks; nonetheless, NYHA/mMRC captured functional burden and remained consistently associated with mortality. These issues mirror systemic under-representation of non-European ancestries in PF genomics and highlight the value of integrating genomic ancestry and broader panels or polygenic scores in future work [6,32].

In summary, *MUC5B* rs35705950 showed a strong association with susceptibility in this Brazilian cohort, whereas functional impairment (NYHA/mMRC) was the most consistent prognostic signal. Integrating genetic information with clinical assessment, and expanding multicenter studies in admixed populations with formal ancestry adjustment, will be key to robustly testing genotype–prognosis relationships and refining risk stratification [6,33].

## Supporting information

S1 Table Genotypic and allelic distributions of the MUC5B rs35705950 (G > T) variant in idiopathic pulmonary fibrosis (IPF) cases and controls under different genetic models. (DOCX)

S1 Dataset Raw anonymized dataset used for analyses. Individual-level data on demographic, clinical, and genotypic variables used in all statistical analyses.
(XLSX)

S1 Checklist STROBE Statement checklist for cohort studies. Completed checklist for observational studies, in compliance with STROBE guidelines.
(XLSX)
