# Supplementary material for: MUC5B rs35705950 and its association with survival in Brazilian patients with idiopathic pulmonary fibrosis: A longitudinal cohort study
Source: PLoS One. 2026 Jan 29;21(1):e0341661. doi: 10.1371/journal.pone.0341661 (PMC12854480; doi:10.1371/journal.pone.0341661)
Supplement: S1 Table — (DOCX) [file pone.0341661.s001.docx]

**Supplementary Table 1:** Comparative analyses of genotypic and allelic distributions for the *MUC5B* rs35705950 G>T variant between the case and the control groups, according to different genetic models.

| **Genetic models** | **Genotype** | **Controls**  **n (%)** | **Cases**  **n (%)** | **Cases vs Controls**  **p /OR (IC 95%)** |
| --- | --- | --- | --- | --- |
| Codominance | G/G | 40 (83.33) | 9 (17.65) | ref |
|  | G/T | 8 (16.67) | 36 (70.59) | <0.0001 / 20.0 (6.97-57.35) |
|  | T/T | 0 (0) | 6 (11.76) | * |
| Dominance | G/G | 40 (83.33) | 9(17.65) | ref |
|  | G/T + T/T | 8 (16.67) | 42 (82.35) | <0.0001 / 23.3 (8.19-66.42) |
| Recessive | G/G + G/T | 48 (100,0) | 45 (88.24) | ref |
|  | T/T | 0 (0) | 6 (11.76) | * |
| Overdominance | G/G + T/T | 40 (83.33) | 15 (29.41) | ref |
|  | G/T | 8 (16.67) | 36 (70.59) | <0.0001 / 12.0 (4.55-31.62) |
| Allele | G | 88 (91.67) | 54 (52.94) | ref |
|  | T | 8 (8.33) | 48 (47.06) | <0.0001 / 9.77 (4.29-22.23) |

*Comparisons indicated by an asterisk (**) were omitted as one of the values was zero, preventing valid statistical analysis.
